# An interesting case of familial chylomicronemia syndrome in a cleft palate child

**DOI:** 10.4103/0970-0358.41116

**Published:** 2008

**Authors:** H. S. Adenwalla, P. V. Narayanan, C. J. Rajshree, Rati Santhakumar

**Affiliations:** Department of Plastic Surgery, Charles Pinto Centre for Cleft Lip, Palate and Craniofacial Anomalies, Jubilee Mission Medical College and Research Institute, Trichur - 680 005, Kerala, India; 1 Department of Pediatrics and Burns, Charles Pinto Centre for Cleft Lip, Palate and Craniofacial Anomalies, Jubilee Mission Medical College and Research Institute, Trichur - 680 005, Kerala, India

**Keywords:** Familial hyperlipoprotenemia, lipoprotein lipase

## Abstract

Familial chylomicronemia syndrome is a very rare condition with an incidence of one in one million. We report such a condition detected incidentally in a cleft child.

## INTRODUCTION

Familial chylomicronemia usually manifests in childhood at around ten years of age with recurrent abdominal pain and pancreatitis. This disorder is usually clinically silent and hence not discovered till blood is sampled for some other reason. During sampling, massive elevation of triglycerides (1,000 mg/dl–>10,000 mg/dl) is noted. In our asymptomatic case, the diagnosis was made during surgery.

## CASE HISTORY

**Figure 1:**
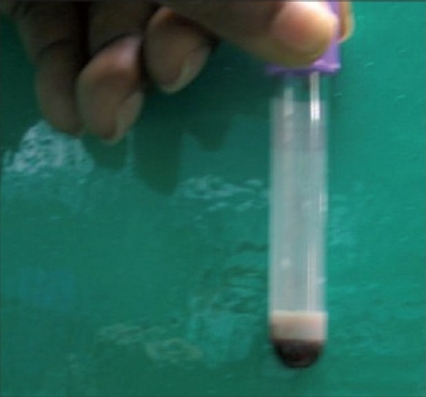
Appearance of centrifuged blood – plasma appears lipid laden

**Figure 2:**
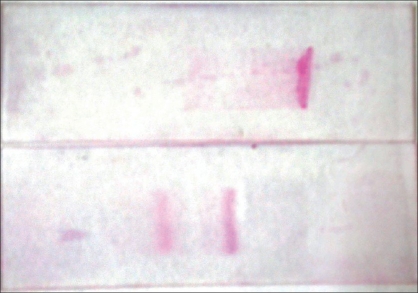
Picture of blood electrophoresis: Chylomicrons appear dominant

A fifteen month-old male child who had undergone cleft lip repair at five months of age and in whom nothing abnormal was found at that stage, was later taken up for cleft palate repair. Peroperatively at the incision sites, the blood was found to be milky [Figure 1]. Blood drawn by venepuncture.was also of a similar nature. This was then sent for biochemical analysis [Figure 2]. The results are shown in Table 1.

**Table 1:** Plasma cholesterol and triglyceride mean level and percentile levels

	*Total triglyceride level (mg/dl)*					*Total cholesterol level (mg/dl)*				
1-4 years	5^th^	Mean	75^th^	90^th^	95^th^	5^th^	Mean	75^th^	90^th^	95^th^
Normal male	29	56	68	85	99	114	155	170	190	203
Normal female	34	64	74	95	112	112	156	173	188	200
This child	Triglyceride 7000 mg/dl					Cholesterol 495 mg/dl				

Level of triglycerides >95^th^ percentile genetic forms of hyperlipdemia

Further investigations done gave the following results:

1. Serum albumin4 gms/dl2. High density lipoprotein (HDL)23 mg/dl3. Urine sugarNil4. Urine proteinNil5. Triiodothyronine (T3)1.02 ng/dl6. Thyroxine (T4)9.6 *µ*g/dl7. Thyroid-stimulating hormone (TSH)0.80 *µ*IU/ml8. Blood urea50 mg/ml1^st^ sample19 mg/ml2^nd^ sample2 days later9. Serum creatine1 mg/dl1^st^ sample0.3 mg/dl2 ^nd^ sample2 days later10. Growth factor receptor-bound blood sugar (GRBS)84 mg/dl

A diagnosis of familial chylomicronemia was made. Post-operatively the child was put on a low-fat diet and advised medium chain triglycerides in the form of coconut oil. The palate has healed well and the child has been discharged home uneventfully.

## DISCUSSION

Of the various Familial Dyslipoprotenemias, Type I Hyperlipoprotenemia or Familial Chylomicronemia is very rare and is autosomal recessive.[1] It is characterized by marked hyperchylomicronemia and a corresponding hypertriglyceridemia (triglyceride as high as 10,000 mg/dL). It may be due to the deficiency of Lipoprotein Lipase (LPL) activity or deficiency of Apolipoprotein C-II. Lipoprotein Lipase is essential for the hydrolysis of triglycerides and the conversion of chylomicrons to chylomicron remnants. The massive accumulation of chylomicrons in the circulation indicates the inability to catabolize dietary fat. The concentration of very low density lipoprotein (VLDL) cholesterol is usually normal.

This disorder is usually expressed in childhood. It appears that those patients with low to absent LPL activity in all tissues present with the disease at an early age (classic form) whereas those with a deficiency of LPL activity in only one tissue become symptomatic later in life (variant form). This disease is usually detected after recurrent episodes of severe abdominal pain and repeated attacks of pancreatitis.[2] The possible mechanism of pancreatitis is the lipolysis of triglycerides that produces high concentrations of free fatty acids in the vicinity of the pancreas which damage small vessels and produce ischemic injury; they may also be toxic to the cell membranes of the acinar cells.

Eruptive xanthomas and lipemia retinalis are usually present when the plasma triglyceride concentrations exceed 2000 and 4000 mg/dL respectively.[3] Xanthomas are painless skin lesions on the back, buttocks and extensor surfaces of the arms and legs. The acuteness of the symptoms is directly proportional to the degree of hyperchylomicronemia. It is important to note that patients with this disorder do not appear to be predisposed to atherosclerotic disease.

Chylomicronemia can also cause neurological manifestations and dyspnea.[4] Accumulation of triglycerides in reticuloendothelial cells can cause hepatosplenomegaly.

LPL deficiency is suspected in any person who has a lipemic serum after a 12 hour fast. Fasting plasma is turbid and if left at 4°C for a few hours, the chylomicrons float to the top and form a creamy supernatant.

Many secondary causes of this condition have been reported [5] and are listed in Table 2.

**Table 2:** Secondary causes of chylomicronemia

Nephrotic syndrome
Hypothyroidism
Renal failure
Storage disease
Systemic lupus erythematosus
Diabetes mellitus
Congenital biliary atresia
Excessive alcohol intake
Drugs-oral contraceptive pills, thiazide diuretics

The diagnosis of chylomicronemia is made by the determination of LPL activity in plasma after an intravenous administration of heparin. Heparin binds with LPL causing its dissociation from heparan sulfate present on the surface of endothelial cells with its subsequent release into plasma.

Patients with a deficiency of Apolipoprotein C-II, the required activator for LPL have similar clinical symptoms but they are usually milder and expressed at a later age. Those affected have less than 10% of the normal concentration of Apo C-II, the minimum amount needed for normal LPL activity. Diagnosis is by the documentation of low LPL activity in postheparin plasma and the restoration of normal enzymatic activity by addition of normal Apo C-II to the assay mixture. Another diagnostic method is by immunoassay for Apo C-II.

As far as long term management is concerned, these patients are placed on a very low fat diet (10-15 g/day in a child). As medium chain triglycerides are absorbed directly from the portal vein, they are used to replace some of the dietary long chain fats for these patients. In cases where fasting triglyceride concentration is over 1000 mg/dL and the patient is already on a fat restricted diet, drug therapy is recommended to reduce the risk of development of pancreatitis.
